# Theft-Safe Explosive Mixtures Based on Hydrogen Peroxide: Study of Properties and Built-In Self-Deactivation Kinetics

**DOI:** 10.3390/ma14195818

**Published:** 2021-10-05

**Authors:** Mateusz Polis, Karolina Nikolczuk, Andrzej Maranda, Agnieszka Stolarczyk, Tomasz Jarosz

**Affiliations:** 1 Explosive Techniques Research Group, Łukasiewicz Research Network—Institute of Industrial Organic Chemistry, 42-693 Krupski Młyn, Poland; mateusz.polis@ipo.lukasiewicz.gov.pl; 2 Łukasiewicz Research Network—Institute of Industrial Organic Chemistry, 03-236 Warsaw, Poland; andrzej.maranda@ipo.lukasiewicz.gov.pl; 3 Department of Physical Chemistry and Technology of Polymers, Silesian University of Technology, 44-100 Gliwice, Poland; agnieszka.stolarczyk@polsl.pl

**Keywords:** explosive, green, secure, hydrogen peroxide, ammonium nitrate, aluminium, detonation velocity, blast wave pressure, Raman spectroscopy

## Abstract

The current focus on both environmental and general safety is an important issue in the field of explosives. As such, environmentally-friendly explosives, based on hydrogen peroxide (HTP) as an oxidising agent, are of significant interest. These explosives can be designed to undergo self-deactivation, denying access to them by any unlawful third parties that may attempt scavenging blasting sites for any residual energetic materials. Such deactivation also improves blasting safety, as, after a set time, misfired charges no longer pose any explosive threat. In this work, we have designed HTP-based explosive formulations that undergo deactivation after approximately 12 h. To this effect, Al powders were used both as fuels and HTP decomposition promoters. The shock wave parameters and ability to perform mechanical work of the proposed explosive formulations are comparable to those of dynamites and bulk emulsion explosives, and the details of the changes of these parameters over time are also reported.

## 1. Introduction

The detonation of classical nitrate- or nitroester-based explosives often results in copious amounts of nitrogen oxides (NOx) being formed, which poses a significant hazard to personnel, particularly in underground blasting operations [1,2]. Even though the nitrogen oxides produced during detonation can be absorbed to an acceptable extent via supplementing the explosive charges, e.g., with urea [3], such supplementation involves a trade-off between reduced NOx formation and explosive power. The amount of nitrogen oxides generated can also be reduced with the addition of aluminium dust (to ammonium nitrate-based explosives) [4,5]; however, this implies a change in all the properties of the high-energy material. Consequently, energetic alternatives to nitrogen-bearing molecules have been sought.

Concentrated hydrogen peroxide (often referred to as either “high-test hydrogen peroxide” (HTP) or “rocket-grade hydrogen peroxide”) is one such alternative. HTP has a history of being used as an energetic material (e.g., its use as a propellant component of V2 rockets) [6] and it has been investigated, in terms of the explosive hazard it poses, e.g., in mixtures with alcohols [7] or water and glycerine [8]. Interestingly, despite true explosive charges utilising HTP as a key energetic component having been developed quite some time ago [9], there has been relatively little work on them in the following years [10], with researchers returning to the subject [11] and conducting comprehensive testing of such charges only recently [12].

The works dedicated to the use of HTP in explosive charges have largely focused on preventing NOx formation, as well as the “green” nature of HTP, being produced mainly in the fairly environmentally-friendly anthraquinone process [13,14] and yielding water and oxygen upon its decomposition. Since HTP does not contain nitrogen, replacing nitrogen-bearing oxidising agents with HTP, either partially or completely, leads to reducing the amount of nitrogen oxides generated by the detonation of such formulations [11].

Another trend in the use of HTP in explosive charges is to produce its adducts with more typical high explosives (HEs), such as 2,4,6,8,10,12-hexanitro-2,4,6,8,10,12-hexaazaisowurtzitane (CL-20) [15] or with ammonium cyclopentazolate [16]. In such adducts, the nitrogen content is reduced, in comparison to those explosives by themselves, resulting in reduced NOx emissions. Simultaneously, the energetic properties of such HTP-HE adducts are reported to be improved, likely due to their increased oxygen balance.

Although the focus on minimising the amount of harmful substances produced during detonation is perfectly valid, it caused both other researchers and ourselves [17] to pass over another, possibly even more crucial feature of HTP-based explosive charges: the gradual decomposition of HTP within these charges. The decay of HTP is both disadvantageous (limiting the time frame, in which the charges need to be used after being prepared) and advantageous (a charge that was “misplaced” or misfired during the blasting operation will eventually lose its capability of undergoing detonation).

Consequently, both the threat of such charges being used by parties that obtained them unlawfully and the hazards related to a misfired explosive charge remaining after blasting can be largely alleviated, if not completely avoided, due to this “disadvantageous” decomposition. Incidentally, despite HTP decomposition being well-known to take place in numerous systems (and even being used to decompose nitrogen-bearing explosives [18]), the rate of its decomposition in explosive charges has not been investigated to date. In light of this, we have undertaken the investigation of the kinetics of HTP decomposition in a fairly standard explosive charge formulation, as well as in a formulation supplemented by aluminium powder. In the current work, we have followed both the HTP decomposition process (using spectroscopic methods) and observed its effects on the performance of explosive charges (focusing on the velocity of the detonation, as well as the ability of the charge to perform mechanical work) over time.

## 2. Materials and Methods

### 2.1. Materials

The on-site mixture matrix (**OSM-0**) was prepared utilising the following chemicals:

- Hydrogen peroxide solution (analytical grade, EnvoLab Chemicals, Dlugomilowice, Poland), with a concentration of 60% by weight, which was diluted with with deionised water to a concentration of 52% by weight and will be referred herein as HTP. It should be noted that this reagent is not available to the general public, as per the relevant laws on the distribution of explosives precursors.
- Ammonium nitrate (V) (technical grade, Nitrogen Plant “Pulawy”, Pulawy, Poland), which was dried at 60 °C for 24 h prior to use.
- Glycerine (pharmaceutical grade, ≥99.5%, TechlandLab, Tarnobrzeg. Poland),
- Guar gum S.C.-406 (Meyhall Chemical AG, Kreuzlingen, Germany), acting as a viscosity modifier.

In order to produce derivative explosive mixtures, the **OSM-0** matrix was first supplemented by aluminium powder, in the form of either aluminium flakes (BLITZ ALUMINUM DEPUVAL 3083 flake aluminum, average particle size 12 μm, bulk density 0.4 g/cm^3^, active aluminium content 82.5%, without protective coating) or atomised aluminium (BLS0052:AG45-90/99.7 ALATOMIZED: FU03, grain size 45–90 μm bulk density 0.9 g/cm^3^, active aluminum content 99.7%, without protective coating), with both powders being used as received (Benda-Lutz, Nußdorf ob der Traisen, Austria). The mixtures supplemented by aluminium flakes and atomised aluminium are denoted as **OSM-Al_f_** and **OSM-Al_A_**, respectively. Next, glass microspheres (type K-015 (3M, Saint Paul, MN, USA)) were added to both the two types of Al-supplemented samples and the OSM-0 matrix. Samples intended for Raman spectroscopy measurements were further supplemented with titanium(IV) oxide anatase (Sigma Aldrich, St. Louis, MO, USA), which was employed as an internal reference substance. The morphology of aluminium powders was studied using a scanning electron microscope (SEM) Phenom Pro-X (Thermo Fisher Scientific, Waltham, MA, USA).

### 2.2. Preparation of On-Site Mixture Samples

First, HTP was placed in the chamber of a DRAIS-type mixing machine, along with ammonium nitrate(V). The two were subjected to mixing at room temperature until the nitrate dissolved completely. Next, glycerine and guar gum were added, followed by a 10 min mixing and homogenisation cycle. For the **OSM-Al_f_** and **OSM-Al_A_** samples, the respective type of aluminium powder was then added to the mixture. Next, glass microspheres were added and gently mixed, so as to minimise microsphere breakage. Formulations of the investigated OSM samples are given in Table 1.

The prepared OSM samples were loaded into previously prepared polypropylene tubes (length of 250 mm, internal diameter of 46.4 mm and wall thickness of 1.8 mm), which were sealed on one end and had series of holes on their sides for equipping them with probes for velocity of detonation measurements. The significant difference in charge density, depending on the aluminum used, is due to charge aeration by flake aluminum. This is due to its large specific surface area and its influence on the structure of the liquid explosive. Once the OSM sample was loaded into the tube, the other end of the tube was sealed and equipped with an axially placed 1,3,5,7-tetranitro-1,3,5,7-tetrazocane (HMX) booster, flegmatised with 5% by weight of a fluoropolymer binder.

**Figure 1:**
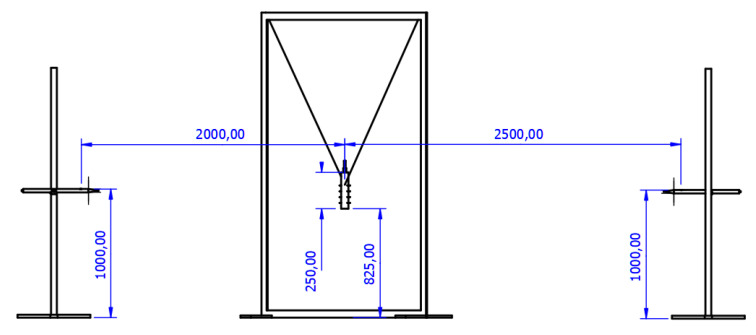
Experimental setup for the determination of shock wave parameters and detonation velocity.

Boosters (mass of the booster = 16 ± 0.2 g), as depicted in Figure 1, were produced by pressing in a steel matrix, under the pressure of 4 MPa. Each of the used boosters was cylindrical in shape (diameter of 21 ± 0.02 mm, height of 30 ± 0.02 mm), with a detonator well (diameter of 8 ± 0.05 mm, depth of 18 ± 0.05 mm) located in the axis of the booster. The density of boosters was 1.68 ± 0.01 g/cm^3^.

### 2.3. Raman Spectroscopy

Raman spectroscopy was performed using a Raman microscope (inVia Renishaw, Wotton-under-Edge, UK), which was equipped with a CCD detector and using green (514 nm) laser excitation. Spectra were recorded in a fixed range of 100–3500 cm^−1^. All measurements were made in a backscattering geometry using a 50× microscope objective with a numerical aperture value of 0.75, providing scattering areas of 1 μm^2^. Single-point spectra were recorded with 4 cm^−1^ resolution and 10 s accumulation times.

**Figure 2:**
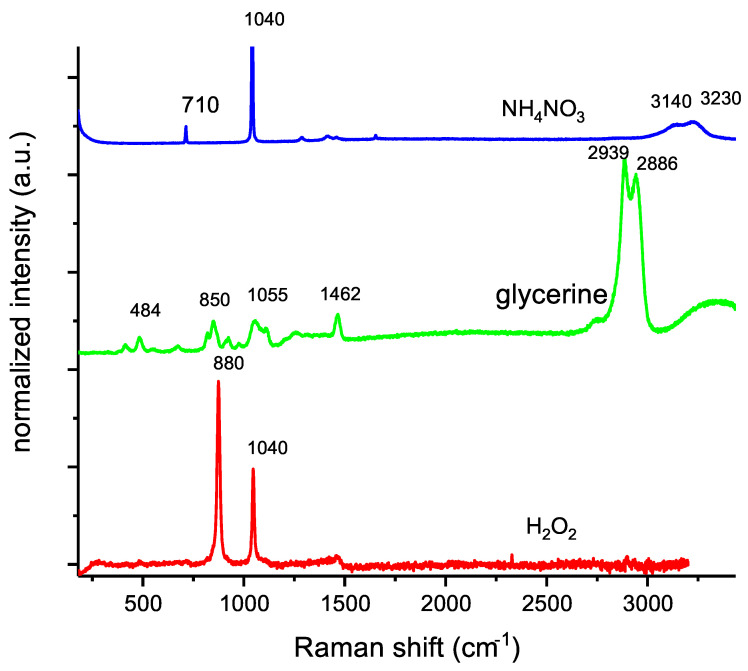
Raman spectra of OSM components.

Figure 2 shows the Raman spectra of the components of the investigated OSM samples. The choice of parameters resulted in no noticeable signals observed in the spectrum acquired for guar gum; therefore, this spectrum is not included in Figure 2. Raman bands, which were characteristic of ammonium nitrate, were found at:

- 710 cm^−1^—in-plane deformation of the NO group (NO_3_^−^).
- 1040 cm^−1^—symmetric stretch of N=O (NO_3_^−^).
- 1287–1550 cm^−1^—vibrational coupling between the ammonium ion deformation and nitrate stretching.
- 3150–3230 cm^−1^—anti-symmetric and symmetric NH stretching.
- 1400 and 1500 cm^−1^ rocking/twisting vibration of the NH_3_^+^ group.

For glycerine, signals were recorded, respectively, at:

- 484, 414 cm^−1^—CCO rocking.
- 820, 850 cm^−1^—C-C stretching.
- 923, 975 cm^−1^—CH_2_ rocking.
- 1055 cm^−1^—C-O stretching.
- 1462 cm^−1^—CH_2_ deformation.
- 2939, 2886 cm^−1^—CH stretching anti-symmetric and symmetric.

In the case of hydrogen peroxide, the peak at about 880 cm^−1^ was assigned to the symmetric vibration between O–O in hydrogen peroxide molecules. The smaller peak, at about 1409 cm^−1^, corresponds to the symmetric vibration between the hydrogen and oxygen atom in both water molecules and hydrogen peroxide molecules.

#### Time-Resolved Raman Spectroscopic Measurements

The decomposition of hydrogen peroxide was observed in the OSM samples at room temperature using Raman spectroscopy. The analytical signal peaked at 880 cm^−1^, corresponding to the symmetric vibration between O–O in the hydrogen peroxide molecule. The three types of OSM samples were supplemented with 0.25% TiO_2_, acting as an internal standard. Consequently, all spectra were recorded in dark conditions, so as to prohibit any kind of photochemical reactions involving TiO_2_. The samples were then homogenised and placed in the Raman spectrometer. The procedure of preparing the OSM samples and calibrating the spectrometer required approximately 10 min. Consequently, the first recorded Raman spectrum for each sample is listed at t = 10 min, with the following spectra being recorded until 6 h (investigation of small-scale OSM samples) or 360 h (investigation of bulk OSM charges) have elapsed in total. Each spectrum was recorded as a single scan, ranging from 300 to 3500 cm^−1^. The measurement results were standardised to the TiO_2_ signal at 616 cm^−1^.

### 2.4. Determination of the Ability to Perform Mechanical Work

The ability of the OSM charges to perform mechanical work was evaluated, in comparison to 1,3,5-trinitro-1,3,5-triazinane (RDX), using a ballistic mortar. Examination of the ability to perform mechanical work using the ballistic mortar relies on the detonation of a fixed mass of the investigated sample to produce force that moves the mortar out of equilibrium. The maximum angle to which the mortar was inclined during the test is a measure of the ability of the sample to perform mechanical work. This method is a relative method and, as such, the results are expressed as a fraction of the mechanical work produced by a reference charge. In our experiments, RDX explosive charges were used, as is most common practice. The reference RDX charges were prepared by placing 10 g of RDX powder (density equal to 1.00 g/cm^3^) into a cellophane casing. Tested charges were prepared by placing 10 g of each OSM charge into a cellophane casting. The samples were initiated by using a detonator equivalent to a standard no. 8 primer, without a booster, according to the PN-C-86035:1999 standard. The experiments were repeated three times for both the reference charges and the investigated OSM samples. The calculation of the ability to perform mechanical work is based on Equation (1).

(1) $$x=\frac{m}{m_{W}}\cdot 100\%$$

where:

- *x*—ability to perform work.
- *m*—arithmetic average of (1−cos(α)) of tested material charges.
- *m_W_*—arithmetic average of (1−cos(α)) value of reference charges.

### 2.5. Determination of Shock Wave Parameters and Detonation Velocity

In order to determine the parameters of the shock wave generated by the investigated OSM charges, two type 137A23 piezoelectric pressure sensors (PCB PIEZOTRONICS) were used. The tested charges were suspended 1 m above the ground from the middle of the load length. The pressure sensors were placed 2 and 2.5 m from OSM charges and 1 m above the ground. The distance between the test loads and the pressure sensors was determined between the axis of the cylindrical charge and the axis of the active element of the pressure sensor. Sensor data were recorded using a digital multichannel MS05104 oscilloscope (Rigol), capable of sampling data at a frequency of 8 Gsa/s. The accuracy of the measurement was limited mostly by the sensitivity of the pressure sensors (14.5 mV/Pa ± 15% and sampling of oscilloscope (1 MSa/s). The detonation velocity values were recorded simultaneously, through the electrical method, using short-circuit probes and a digital multichannel MS05104 oscilloscope. Four probes were placed crosswise to the axis of the charge, at a separation of 40 mm between neighbouring probes. The distance between the first probe and booster (which was employed, due to being necessary for stabilising the velocity of detonation) was equal to 90 mm. The detonation velocity was calculated based on measured time and the distance between the probes. The accuracy of the detonation velocity measurement was limited by the sampling rate of oscilloscope (1 MSa/s) and precision of probes placement (±1 mm) The measurements were conducted at set time intervals after the preparation of the bulk (500 g) OSM charges, in order to monitor the planned decay of their properties, caused by the gradual decomposition of hydrogen peroxide. The schematic of the stand is shown in Figure 1.

**Figure 3:**
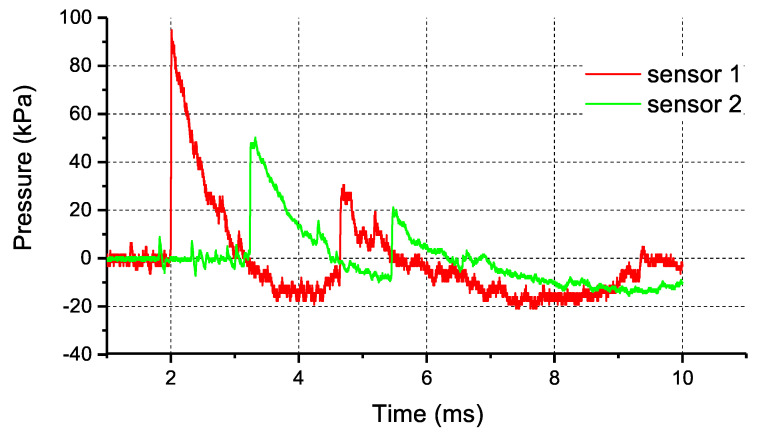
Sample pressure vs. time profiles for the detonation of OSM-0 charges.

Typical pressure–time characteristics for OSM charges are shown in Figure 3.

## 3. Results and Discussion

### 3.1. Decay of Hydrogen Peroxide in OSM Samples

Due to the expected on-going decomposition of HTP in the charges (Figure A6, Appendix A), measurements of both the density (Figure A5, Table A1) of OSM samples, and their velocity of detonation have been conducted as a function of time. In all cases of testing small-scale samples, after 12 h of conditioning, no detonation was observed for those charges.

**Figure A4:**
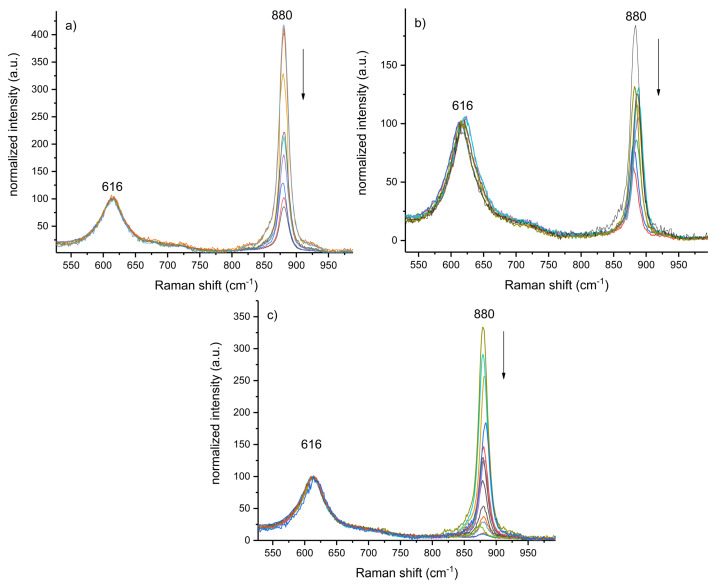
Raman kinetic spectra obtained by measurement of samples (**a**) OSM-0, (**b**) OSM-Al_f_, (**c**) OSM-Al_A_, (**c**) Signal changes at 880 cm^−1^—symmetric vibration between O–O in hydrogen peroxide molecule as a function of time for tested OSM. The arrow indicates the direction of the signal changes.

**Figure A5:**
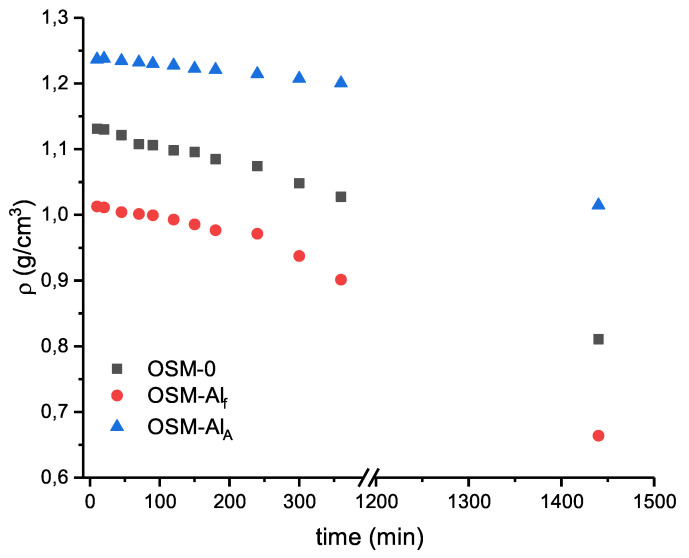
Evolution of OSM density over time elapsed after their preparation.

The time-resolved Raman spectra (Figure A4 of the small scale (10 g) OSM samples shows the gradual decay of the signal at 880 cm^−1^, attributed to hydrogen peroxide. Although the shapes of the two observed signals differ slightly between the three types of OSM formulations, the decay of this signal is observed in each case, confirming that HTP decomposition is taking place in the samples.

**Figure 4:**
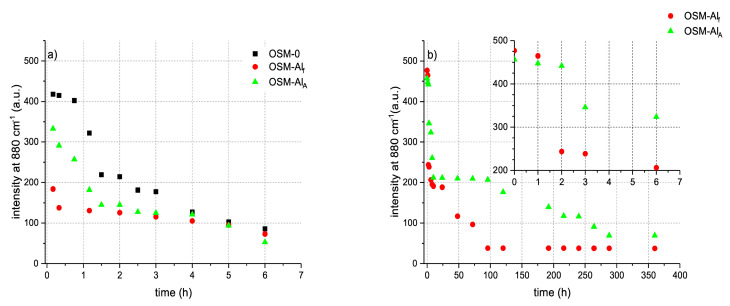
Evolution of the signal at 880 cm^−1^. Symmetric vibration between O–O in hydrogen peroxide molecules, as a function of time for tested OSM for (**a**) 10 g OSM and (**b**) 500 g OSM; Inset: magnification of signal changes for 500 g OSM charges, taking place over the first 6 h of the experiment, for comparison with panel a.

Interestingly, despite the HTP decomposition taking place in all three types of OSM samples (Figure 4a), it is worth noting that for OSM-0, the decomposition is initially only minor, appearing to be induced and begin accelerating after approximately 1 h has elapsed. Such an induction period is not observed in the case of either of the aluminium-supplemented samples (OSM-Al_f_ and OSM-Al_A_). Among the two aluminium-supplemented samples, the decomposition is more rapid for OSM-Al_f_. After approximately 4 h have elapsed, however, the concentration of HTP in all three types of OSM samples becomes similar and continues to gradually decrease.

The initial decay induction period for OSM-0 can be the symptom of an autocatalytic reaction, where the initial products of oxidation of the organic components (glycerine, guar) by HTP promote the decomposition of HTP. The lack of such an induction period, in the case of OSM-Al_f_ and OSM-Al_A_, supports this hypothesis, as both Al and Al_2_O_3_ are known to promote the decomposition of HTP [19,20,21,22], acting either as reducing agents or as catalysts.

The difference in the decomposition rates of HTP in OSM-Al_f_ and OSM-Al_A_ can be explained by the fact that aluminium flakes have a significantly higher specific surface area than atomised (spherical) aluminium particles. Consequently, the larger surface area of aluminium available for reaction with HTP in OSM-Al_f_ than in OSM-Al_A_ translates into a higher HTP decomposition rate.

The kinetics of decomposition were also investigated for bulk OSM charges (500 g). Due to the relatively slow decomposition of OSM-0 and their lower performance parameters (Table 2), only aluminium-supplemented OSM samples were investigated in bulk.

The transition from small samples (10 g) to bulk OSM charges (500 g) significantly decelerates the decomposition of HTP (Figure 4b). Although the trend of HTP decomposition is similar, i.e., OSM-Al_f_ shows a more rapid decomposition than OSM-Al_A_; the shape of the decomposition curve differs significantly from that observed for small-scale samples.

**Figure A6:**
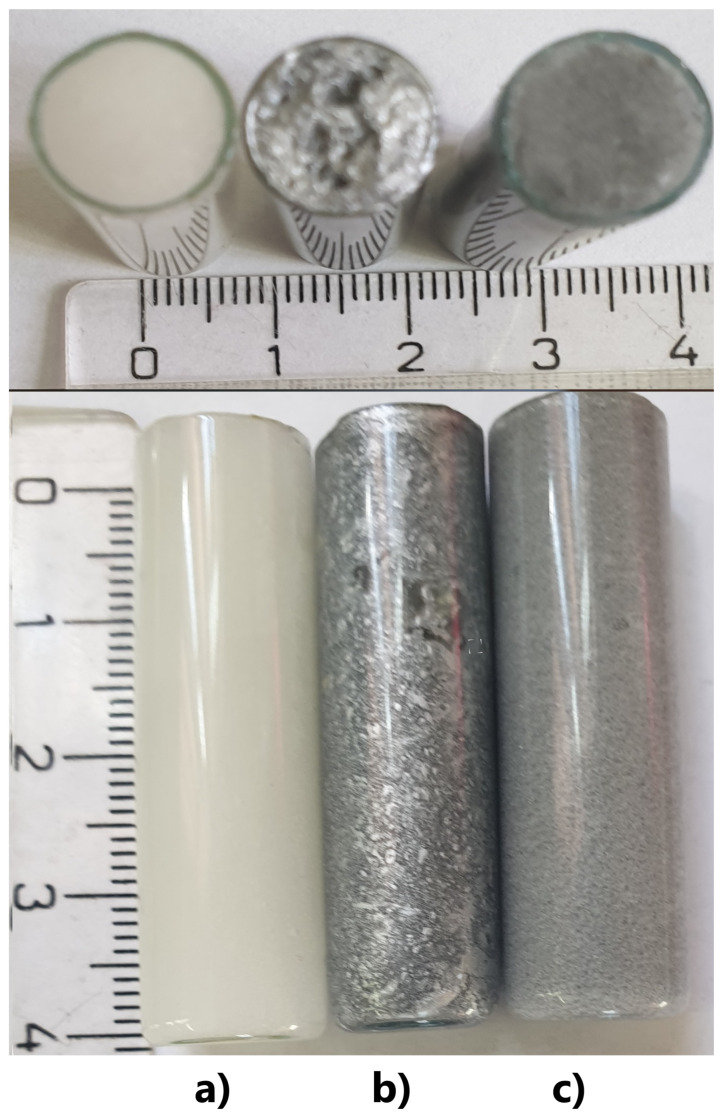
Photographs of OSM samples taken when 24 h have elapsed after their preparation (**a**) OSM-0, (**b**) OSM-Al_f_, (**c**) OSM-Al_A_.

The slower decomposition of HTP in the bulk charges can be attributed to the fact that oxygen, which is one of the HTP decomposition products, needs to evacuate from the sample, in order to affect the equilibrium of the decomposition reaction. In small-scale samples, such an evacuation takes place readily, due to the small dimensions of the samples and relatively large open surface-area-to-volume ratio. In the case of the bulk charges, such an evacuation is hindered, particularly from within the bulk of the charge, resulting in oxygen bubbles forming within the OSM matrix and further hindering oxygen evacuation from the charge. Such an effect was even observed in the small-scale samples, although to a much lesser extent than in the bulk charges, with cavities forming in the samples (Figure A6).

### 3.2. Determination of Ability to Perform Mechanical Work

The produced OSM charges show an acceptable ability to perform mechanical work, with an increase of aluminium content, translating to an increased ability to perform mechanical work. This ability is even greater for OSM-0 than what is reported in literature for ammonium nitrate (V)/fuel oil (ANFO) explosives. The aluminium-supplemented formulations are comparable in this aspect, even with the performance of bulk emulsion explosives and dynamites (Table 2).

OSM-Al_a_ shows higher ability to perform mechanical work than OSM-Al_f_, with the same Al content. Simultaneously, for OSM-Al_a_, increasing the Al content from 9% to 13.5% yields only a minor increase in the ability to perform mechanical work, whereas in the case of OSM-Al_f_, significant improvement is observed.

The better performance of OSM-Al_a_ and its “saturation” with Al stem, from the fact that, upon contact with HTP, Al undergoes oxidation to Al_2_O_3_. This oxide seals the surface of the Al particles, effectively preventing HTP from reacting with the Al in the bulk of the particles, even though decomposition of HTP takes place on Al_2_O_3_ [22]. Upon initiation of the OSM charges by the explosive booster, the high pressure results in fracturing the Al_2_O_3_-coated particles, revealing unreacted Al, allowing it to take place in the reaction.

The amount of this unreacted Al is dependent on both the particle size and their ratio of surface-area-to-particle volume. This ratio is significantly lower for atomised aluminium than for flaked aluminium, resulting in a higher fraction of the initial Al content being preserved until initiation, in the case of OSM-Al_A_. The oxidation of this Al will, in turn, improve the energy balance of the detonating OSM, acting synergistically with the higher HTP content, resulting from its slower decomposition for OSM-Al_A_ than for OSM-Al_f_ (Figure 4). With a sufficient fraction of Al being preserved in its unreacted form prior to initiation, increasing its content beyond a certain optimum may adversely affect the performance of OSM-Al_A_, due to the oxygen balance of the formulation becoming increasingly negative and the decomposition of HTP becoming increasingly rapid. Although determining this optimal Al content is a matter for further study, it can be approximated to be below 13.5% and is likely not significantly in excess of 9%. Consequently, further investigations were performed for the formulations, containing 9% Al, that were utilised.

### 3.3. Determination of Shock Wave Parameters

The measured detonation velocity (Table 3) slowly decreases over time for OSM-Al_F_ and is maintained at an almost constant level for OSM-Al_A_. In both cases, although the pressure parameters generally decrease over time, with respect to the initial performance, this decrease in non-monotonic.

The observed diminishing of shock wave parameters over time can, in general, be attributed to the gradual decay of HTP. This decay is expected to be accompanied by phenomena that can, to some extent, mitigate the adverse effect of HTP decay on OSM shock wave parameters, resulting in their observed non-monotonic diminishing.

Among such phenomena, the formation and trapping of oxygen bubbles within the OSM matrix is of particular importance in the case of the investigated bulk (500 g) charges. Not only does it affect the decay of HTP (Figure 4b), but also prevents the loss of a significant fraction of oxygen from the matrix, which helps maintain the oxygen balance of the formulation over time. The formed oxygen bubbles can also sensitise the formulation, acting as a source of “hot spots” in the OSM matrices during their energetic decomposition.

In the case of OSM-Al_f_, where HTP decomposition is fairly rapid and Al is oxidised to a high extent, this mitigating effect manifests as a slight increase of the diminishing impulse and maximum pressure, after approximately 120 min. In the case of OSM-Al_A_, however, due to both higher Al retention and slower HTP decomposition, not only does the increase in impulse and maximum pressure manifest earlier than for OSM-Al_f_ (after approximately 90 min have elapsed), but also results in the initial detonation velocity being roughly maintained over the entire ageing period.

Both the overpressure and the pulse values are lower for OSM-Al_f_ than for OSM-Al_A_. This may indicate the chemical inertia of the flaked Al on the detonation wave front, due to a higher fraction of Al being oxidised, rather than in the case of atomised Al. Simultaneously, the Al_2_O_3_ formed during the oxidation of Al acts as a ballast, consuming heat (due to heating and during its phase transitions), which results in a reduction of detonation parameters. The observed decrease in OSM matrix density (Table A1) should also be taken into account, as it also contributes to diminishing the detonation parameters.

In a situation where the flaked Al is oxidised at the front of the wave, the pressure and impulse would most likely be much higher. The abnormal charge behaviour of flaked Al can be explained not only by the charge structure, but also by the highly developed specific surface of the aluminum, as mentioned earlier.

## 4. Conclusions

Based on the presented experimental results, we can conclude that the atomised Al is the most suitable for OSM formulations based on HTP, as OSM-Al_A_ was found to show the best performance and stability of shock wave parameters during its ageing. The performance of the OSM formulations is, in general, comparable to that of dynamites [23] and bulk emulsion explosives (in the case of OSM-Al_A_) [4], while exceeding the performance parameters reported for other HTP-based explosive formulations [1].

The optimal Al content for these formulations needs to be further optimised, but can be estimated to be on the order of 9%. Although the “built-in HTP decay” mechanism was observed, the time required for the self-deactivation of charges was found to vary significantly with charge size. This can be considered an aspect for future improvement; it would be ideal for the deactivation time to become independent of charge size, implying that the homogeneity of the charges needs to be improved. This can be achieved by optimising the OSM matrix composition.

Simultaneously, this optimisation can also be targeted at the fine-tuning of the charge deactivation time, so that it would be long enough to allow for performing blasting operations without rushing, while short enough to not allow for the theft of the charges. This can be achieved by supplementing the OSM formulations with HTP decomposition catalysts that become active after a set time, e.g., due to their release from a carrier. That can lead to the develop of self-sensitizing materials by partially decomposing the oxidising agent in place of sensitizing the OSM charges, instead of using glass microspheres, after a precise time after mixing, resulting in increased blasting safety.

## Figures and Tables

**Table 1:** Components in based formulation.

Components wt% ^1^	OSM-0	OSM-Al_5_	OSM-Al_10_	OSM-Al_15_
Hydrogen peroxide 52%	72.3	69.0	65.7	62.4
Ammonium nitrate (V)	9.9	9.4	9	8.5
Glycerine	13.8	13.2	12.6	12
Guar gum	3.0	2.9	2.7	2.6
Glass microspheres	1.0	1.0	1.0	1.0
Al_a or f_	-	4.5	9.0	13.5

^1^ Samples for Raman spectroscopic measurements were further supplemented with 0.25% by weight of TiO_2_.

**Table 2:** Relative ability to perform mechanical work, determined via the ballistic mortar method, with RDX as the reference explosive.

Formulation	Al Content (wt. %)	Response (% RDX)	Ref.
OSM-0	0	65.1	-
OSM-Al_f_-5	4.5	70.1	-
OSM-Al_f_-10	9	74.6	-
OSM-Al_f_-15	13.5	79.1	-
OSM-Al_a_-5	4.5	73.7	-
OSM-Al_a_-10	9	84.0	-
OSM-Al_a_-15	13.5	84.7	-
ANFO	-	51	[23]
Dynamite	-	84	[23]
Bulk emulsion explosive	-	80.4	[4]
Bulk emulsion explosive	3	82.5	[4]
Bulk emulsion explosive	7	84.4	[4]

**Table 3:** Summary of the changes of the energetic properties of OSM charges over time elapsed from their preparation.

OSM-Al_f_						
**Time Elapsed (min)**	**Detonation Velocity (m/s)**	**Impulse (Pa∗s)**		**P_MAX_**		**Density (kg/dm^3^)**
		**Sensor 1**	**Sensor 2**	**Sensor 1**	**Sensor 2**	
30	4959	34.78	24.46	92.73	59.53	0.92
60	4681	32.48	21.93	79.89	52.11	0.92
90	4734	32.43	22.68	85.60	56.00	0.91
120	4715	34.57	24.48	91.30	57.79	0.91
150	4563	34.07	22.59	81.32	55.22	0.92
180	4428	34.02	24.09	84.17	54.58	0.92
**OSM-Al_a_**						
**Time Elapsed (min)**	**Detonation Velocity (m/s)**	**Impulse (Pa∗s)**		**P_MAX_**		**Density (kg/dm^3^)**
		**Sensor 1**	**Sensor 2**	**Sensor 1**	**Sensor 2**	
30	5109	41.21	27.54	93.21	50.63	1.14
60	5146	39.54	26.60	92.11	52.39	1.14
90	4958	40.52	26.99	93.81	50.70	1.14
120	5171	40.33	26.32	92.47	46.85	1.14
150	5121	39.11	25.01	85.56	48.82	1.14
180	5218	39.10	27.43	90.96	50.50	1.14

**Figure A1:**
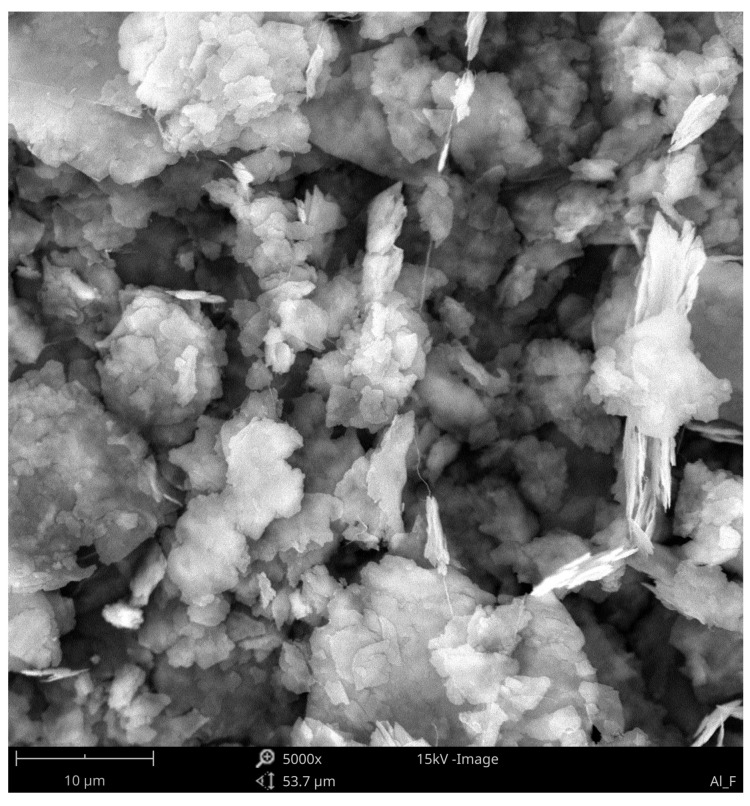
SEM micrograph of flaked aluminium used in this work.

**Figure A2:**
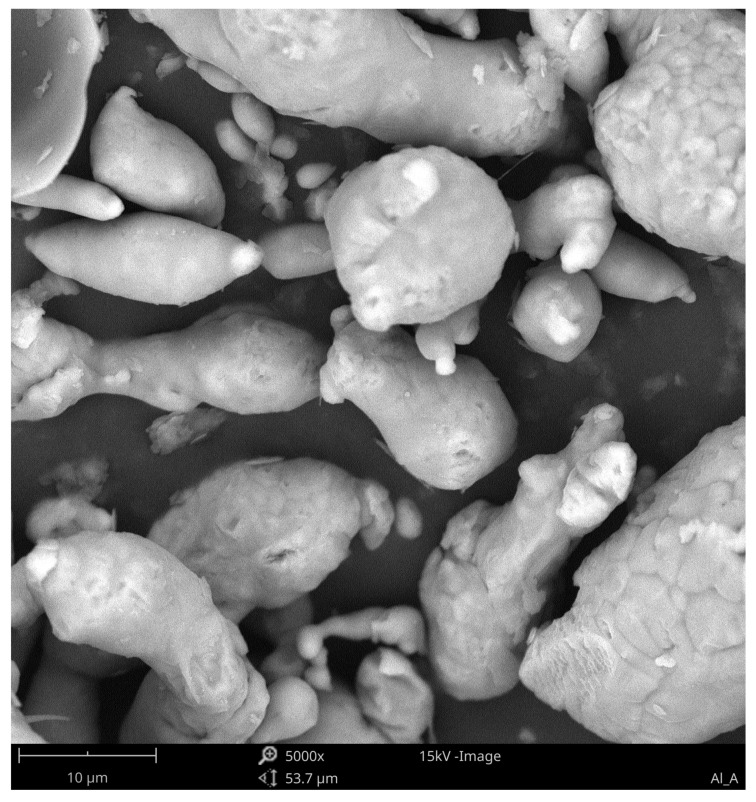
SEM micrograph of atomised aluminium used in this work.

**Figure A3:**
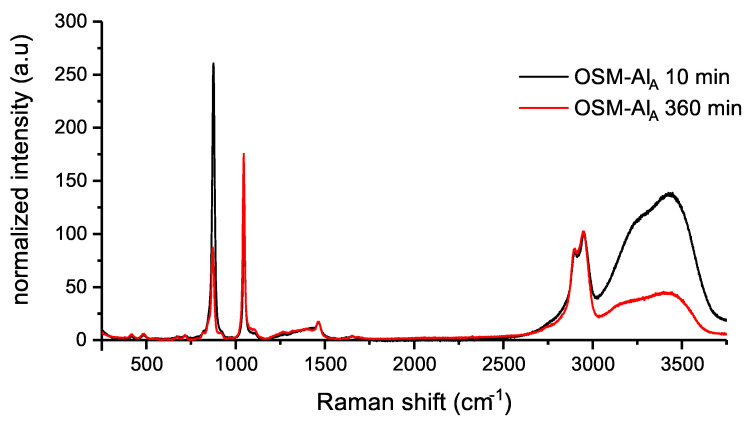
Raman spectra of OSM-Al_A_ in the beginning (10 min—black line) and after 360 min from preparing the material.

## Data Availability

Experimental data sets can be obtained directly from the Authors.

## Appendix A

**Table A1:** Density of OSM samples at selected times after their preparation.

Time	ρ _OSM-0_	ρ _OSM-Al_f__	ρ _OSM-Al_A__
h	g/cm^3^	g/cm^3^	g/cm^3^
as prepared	1.131	1.013	1.166
6	1.028	1.019	1.201
24	0.811	0.664	1.015
